# Effect of Feed Supplemented with Selenium-Enriched Olive Leaves on Plasma Oxidative Status, Mineral Profile, and Leukocyte DNA Damage in Growing Rabbits

**DOI:** 10.3390/ani10020274

**Published:** 2020-02-11

**Authors:** Simona Mattioli, Patrizia Rosignoli, Roberto D’Amato, Maria Chiara Fontanella, Luca Regni, Cesare Castellini, Primo Proietti, Antonia Concetta Elia, Roberto Fabiani, Gian Maria Beone, Daniela Businelli, Alessandro Dal Bosco

**Affiliations:** 1 Department of Agricultural, Environmental and Food Science, University of Perugia, 06121 Perugia, Italy; roberto.damato@unipg.it (R.D.); regni.luca.agr@gmail.com (L.R.); cesare.castellini@unipg.it (C.C.); primo.proietti@unipg.it (P.P.); daniela.businelli@unipg.it (D.B.); alessandro.dalbosco@unipg.it (A.D.B.); 2 Department of Chemistry, Biology and Biotechnology, via del Giochetto, University of Perugia, 06126 Perugia, Italy; patrizia.rosignoli@unipg.it (P.R.); roberto.fabiani@unipg.it (R.F.); 3 Department for Sustainable Process, Agricultural Faculty, Università Cattolica del Sacro Cuore of Piacenza, 29122 Piacenza, Italy; mariachiara.fontanella@unicatt.it (M.C.F.); gian.beone@unicatt.it (G.M.B.); 4 Department of Chemistry, Biology and Biotechnology, University of Perugia, Via Elce di Sotto 8, 06123 Perugia, Italy; antonia.elia@unipg.it

**Keywords:** *Olea europaea*, selenium, blood, oxidative status, minerals, DNA damage

## Abstract

**Simple Summary:**

Olive trees (*Olea europaea* L., Oleaceae) are among the most extensively cultivated crops in the Mediterranean countries (98% of the world’s olive production) and are used for oil extraction or table olives, whereas the residues from pruning (olive leaves and wood) and extraction process (olive pomace) are considered waste or by-products. Furthermore, many agronomic practices, such as foliar spray administration of selenium used to reduce the water stress damage of olive trees, could further improve the nutritional value of these “wastes”. The use of by-products as part of the rabbit diet can be a very effective example of recovery of healthy molecules while at the same time a way of developing a more sustainable production system. Accordingly, the idea of the present research is to administer the by-product “olive leaves” to growing rabbits to improve their health status and partially solve the problem of waste disposal of olive trees.

**Abstract:**

This study investigated the effect of a dietary combination of selenium and olive leaves on rabbit health status in order to evaluate the potential use of these combinations as functional ingredients in feed and food. Sixty weaning rabbits were fed with three diets: control feed (C), control feed + 10% normal olive leaves (OL), or olive leaves enriched in Se (2.17 mg Se/kg d.m.; SeOL). The plasma mineral profile, antioxidant status, and leukocyte DNA damage were determined. Inorganic Se was the most abundant form in the OL diet, while the organic one was higher in SeOL than C and OL. A similar trend was found in the plasma. Protein oxidation showed higher values in both supplemented groups; in addition, dietary Se led to a significant improvement (+40%) in ferric reducing ability of plasma (FRAP). A marked reduction in DNA damage (9-fold) was observed in the SeOL group compared to C. The combination of selenium and olive leaves in the diet of growing rabbits increased plasma SeMet and FRAP and reduced leukocyte DNA damage.

## 1. Introduction

The trace mineral selenium (Se) is an essential nutrient of fundamental importance to human physiology. It is a constituent of at least 25 selenoproteins, mainly in the form of selenocysteine (SeCys_2_) [1]. The uniqueness of Se is also reflected by its biological activity, as this element is often described as a “double-edged sword”, “essential poison”, or “two-faced element” [2]. This is due to its high redox properties; the best-known example of this function is the reduction of hydrogen peroxide and damaging lipid and phospholipid hydroperoxides to harmless products (water and alcohols) by the selenium-dependent glutathione peroxidase family [3]. This function helps to maintain membrane integrity, protects prostacyclin production, and reduces the likelihood of the propagation of further oxidative damage to biomolecules such as lipids, lipoproteins, and DNA with the associated increased risk of atherosclerosis and cancer [4]. Most of the scientific literature related to Se is associated with its antioxidant [2] and anticancer properties [5,6]. The mechanism of its anticancer activity is generally related to both the antioxidant and redox properties of Se, exerted at the nutritional level and linked mainly to the activity of selenium-dependent enzymes (selenoproteins, i.e., SeCys_2_, selenomethionine (SeMet) and selenomethylselenocysteine (MeSeCys)), as well as from its prooxidant effects, observed at the supranutritional level and associated with the activity of low molecular weight Se compounds [7,8]. Indeed, the relatively small difference between the recommended daily intake (70 µg/day in Europe [5]) and the estimated upper tolerable limit (400–800 µg/day according to different sources [9]) should be highlighted.

In animal models, it has been demonstrated that the roles of Se are due to a dose–response relationship; reductions in experimental tumorigenesis are seen when Se is fed at 10–20 times the nutritional level (at least 1.5 mg/kg) [10]. Biochemical and physiological damage due to Se deficiency are prevented by a low dietary level (0.1 mg/kg), whereas adverse effects are reported when more than 5 mg Se/kg diet is used [11]. Furthermore, the positive effect of Se against human cancer (mainly prostate cancer) is often reported in association with antioxidant compounds, mostly with vitamin E [12]. 

In recent decades, numerous scientific publications have highlighted the health effects of plant polyphenols, such as oleuropein, which is particularly present in olive leaves, together with other secoiridoids, flavonoids, and triterpenes [13,14]. Olive leaf extracts have antioxidant, antimicrobial, anti-inflammatory, and anticancer properties and are also able to affect blood pressure, cholesterol, and glucose levels [15,16]. Moreover, in our recent papers, it was demonstrated that Se as a supplemented ingredient (almost 200 µg/kg of feed) in rabbit feed improved the meat oxidative status and provided meat that satisfies the Se requirement of a human adult [17,18]. Similarly, Aouidi et al. [19] added olive leaf powder to beef meat, thereby inhibiting lipid and protein oxidation and also improving the technological quality of the meat. Proietti et al. [20] showed that the dietary intake of Se in sheep can largely offset the effects of oxidative stress on Ca^2+^ homeostasis. As a consequence, some animal-originated products, e.g., meat, milk, and eggs, enriched with selenium have been developed and are currently marketed all over the world [21]. 

Based on these considerations, in the present study, the effect of the dietary combination of selenium and olive leaves on rabbit health status was investigated in order to evaluate the potential use of these combinations as functional ingredients in feed and food. In particular, we investigated the mineral profile of rabbit plasma, the antioxidant status of the blood, and DNA damage rabbit leukocytes as biomarkers of anti-cancer effects. The selenate-treated leaves were derived from pruning olive trees treated with Se in order to reduce tree drought stress [20,22,23]. In this way, in a circular economy logic, the leaves could be used as a Se vector for rabbit feed with the aim of providing an organic source of Se with greater bioavailability [24,25] and at the same time contributing to the disposal of pruning by-products [26,27].

## 2. Materials and Methods

### 2.1. Animals and Diets

The experimental protocol was devised according to Directive 2010/63/EU, transposed to DR 26, on animal welfare for experimental and other scientific purposes [28]. The research was carried out at an experimental farm of the Department of Agricultural, Food and Environmental Science of the University of Perugia (Italy) in the spring of 2015. The experimental design was reported in the previous paper [17]. Briefly, 60 New Zealand white rabbits (35 days old) were allocated in bicellular wire net cages (60 × 25 cm length × 35 cm height) and divided into three homogeneous groups (20 animals/group) and subjected to three different isoenergetic and isonitrogenous dietary treatments (pelleted feed composition: 88.86 g/100 g dry matter, 2.78 g/100 g ether extract, 16.21 g/100 g crude protein, 8.21 g/100 g ash, 14.10 g/100 g crude fiber, 27.15 g/100 g NDF, 18.42 g/100 g ADF, 4.29 g/100 g ADL) until slaughter (for a total of 35 days):

Control feed (C);

Control feed supplemented with 10% olive leaves (OL);

Control feed supplemented with 10% olive leaves enriched in Se (2.17 mg of Se per kg of dried leaves; SeOL).

The choice of rabbit as the animal model was made to trace the possible parallels in biochemical parameters between humans and other animal species, to allow some reliability predictions and extrapolation of the results from one species to another [29].

The olive leaves were obtained as reported in Mattioli et al. [18,30]. Briefly, 30 randomly selected olive trees were sprayed with a water solution containing 100 mg/L of sodium selenite (Sigma-Aldrich, Milan, Italy). The leaves were collected after pruning (November), dried in a ventilated stove at 60 °C for 24 h and subsequently ground, in order to be mixed with the other ingredients of diets [18,30]. 

The feeding program of rabbits was adjusted according to previous studies [18]. Water was supplied *ad libitum*, with the applied temperature and lighting schedules in the rabbit house equal to 15–18 °C and 16 L:8 D, respectively. The feed intake of the rabbits was recorded on a daily basis in order to evaluate the estimated intake of each compound (minerals and antioxidants).

### 2.2. Slaughtering and Blood Sampling

At 70 days of age, 10 rabbits per group were electrically stunned and slaughtered by cutting the carotid artery and jugular veins. Blood samples were collected from each animal immediately after the cutting of the vessels. Five mL blood samples were collected in vacutainers and transported to the laboratory of the Department of Agricultural, Environmental and Food Science at the University of Perugia. Serum was obtained from blood samples coagulated at room temperature for 2 h, and then the collection tubes were rimmed and refrigerated at 4 °C for 24 h before analysis.

Plasma was obtained from blood samples collected into tubes containing Na_2_-EDTA and immediately centrifuged at 2500× *g* for 10 min at 4 °C to determine the hematological parameters. Peripheral blood mononuclear cells (PBMCs) were obtained from heparinized vacutainers.

#### 2.2.1. Isolation of Peripheral Blood Mononuclear Cells

Peripheral blood mononuclear cells were isolated from heparinized vacutainers on a density gradient as previously reported with some changes [31]. Briefly, rabbit blood samples (2 mL) diluted to 8 mL with RPMI 1640 mammalian cell culture media, without serum, were layered over 2 mL of Histopaque 1083 (Sigma-Aldrich, Milan, Italy) and centrifuged at 1600 rpm for 20 min. The layer containing the mononuclear cells at the interface between the plasma and the Histopaque was recovered and washed twice with RPMI 1640. The viable PBMC obtained were counted by the trypan-blue exclusion technique, and the density was adjusted to 1 × 10^6^ cells/mL with complete RPMI (RPMI 1640 with 10% fetal bovine serum, 2.0 mM L-glutamine, 100 U/mL penicillin, and 100 U/mL streptomycin. The PBMCs were then used for the following basal DNA damage analysis.

#### 2.2.2. Mineral Evaluation of Diets and Rabbit Plasma

Feed and plasma samples were analyzed following the method of Mattioli et al. [18] for total Se, Ca, Mg, Fe, Na, K, P, Cu, and Zn contents. 

Se concentrations were determined via ICP-MS (Agilent 7900, Agilent Technologies, Santa Clara, CA, USA) and an Octopole Reaction System (ORS) following the analytical method of D’Amato et al. [20], while the total Ca, Mg, Fe, Cu, and Zn contents were determined using a Shimadzu AA-6200 atomic absorption spectrophotometer (Shimadzu, Tokyo, Japan), as described by Mattioli et al. [18].

#### 2.2.3. Selenium Speciation in Diets and Rabbit Plasma

Diet samples were treated as reported by Mattioli et al. [18]. Quantification of organic and inorganic Se species was performed via HPLC (Agilent 1100, Agilent Technologies, Santa Clara, CA, United States) using an anion exchange column (Hamilton, PRP-X100, 250 × 4.6 mm, 5 μm particle size) with analytical method and instrument parameters described by Bocchini et al. [32]. Results are expressed as ng/mL of plasma.

#### 2.2.4. Blood Oxidative Parameters

The blood lipid peroxidation (thiobarbituric reactive substances, TBARs) was evaluated on plasma using a spectrophotometer (set at 532 nm, Shimadzu Corporation UV-2550, Kyoto, Japan). The 1,1,3,3-tetraethoxypropane calibration curve was prepared in sodium acetate buffer (pH 3.5) [33]. The results are expressed as nmol of malondialdehyde (MDA) per mL of plasma.

The detection of protein carbonyl groups followed the method of Dalle Donne et al. [34], using the 2,4-dinitrofenilhidrazina (DNPH) reactive. The serum was diluted 1:40 with phosphate-buffered saline solution (PBS) before the analysis. Carbonyl contents were determined from the absorbance at 366 nm using a molar absorption coefficient of 22,000 M 1/cm. The results were expressed as nmol/mg of proteins.

The α-tocotrienol, α, γ, δ-tocopherols, and retinol levels of plasma were assessed according to Schuep and Rettenmeier [35] with an HPLC system (Jasco, pump model PU-1580, equipped with an autosampler system, model AS 950-10, Tokyo, Japan) on a Sinergy Hydro-RP column (4 µm, 4.6 × 100 mm; Phenomenex, Bologna, Italy). Tocopherols were identified using a fluorimetric (FD) detector (model Jasco, FP-1520, Jasco, Tokyo, Japan) set at excitation and emission wavelength of 295 nm and 328 nm, respectively, and were quantified using external calibration curves prepared with increasing amounts of pure tocopherols in ethanol. Retinol was identified using a UV–VIS spectrophotometer detector (Jasco UV2075 Plus, Jasco, Tokyo, Japan) set at λ 325 nm and quantified by comparing the sample with a pure commercial standard in ethanol (Sigma-Aldrich, Steinheim, Germany; Extrasynthese, Genay, France).

Glutathione peroxidase activity was measured by following the oxidation of β-nicotinamide adenine dinucleotide (NADPH) at 340 nm and using 1.2 mM H_2_O_2_ as the substrate. The assay condition was as follows: 100 mM NaH_2_PO_4_ + Na_2_HPO_4_ buffer pH7.5, 1 mM EDTA, 0.24 mM NADPH, 2 mM GSH, 1U of glutathione reductase (GR), and 1 mM sodium azide (NaN_3_) [36].

#### 2.2.5. Antioxidant Plasma Power Quantification

The ferric reducing ability of plasma (FRAP) test was used to quantify and compare the antioxidant ability of plasma in control and treated rabbits. This method is based on the measure of the reducing ability of plasma. Electron donor compounds reduce ferric ion (Fe^3+^), in excess in the reaction mixture, to ferrous ion (Fe^2+^) at low pH. The 2,4,6-tripyridyl-s-triazine (TPTZ) in the reaction medium can bind the ferrous ion at low pH. An intense blue color is developed with an absorption maximum at 593 nm:

Fe(TPTZ)^3+^→Fe(TPTZ)^2+^

The color intensity is a measurement of the ferric reducing ability of plasma and, therefore, of its antioxidant capacity. The reagent concentrations were as follows: acetate buffer (300 mM, pH 3.6), TPTZ (10 mM in HCl 40 mM), FeCl_3_·6H_2_O (20 mM) [37].

The FRAP reagent was prepared by mixing 10 volumes of acetate buffer, 1 volume of TPTZ, and 1 volume of FeCl_3_·6H_2_O. Plasma samples were not diluted. In the reaction, 3 mL of FRAP reagent, 100 µL of sample, and 300 µL of deionized water were mixed in a cuvette. This cuvette was shaken, and the absorbance recorded for 8 min at 593 nm. The measurements of absorbance were performed in triplicate. A calibration curve was constructed with aqueous solutions of known Fe^2+^ concentrations (0, 100, 300, 500, 750, and 1000 µmol/L FeSO_4_ 7H_2_O). Results are expressed as µmol Fe^2+^/L.

#### 2.2.6. DNA Damage Assessment

Basal DNA damage was determined in 4 PBMCs rabbit samples per group by the single cell gel electrophoresis assay (SCGE or comet assay), as previously described [38]. For this, 10 µL of a cell pellet, three replicates for each sample, resuspended in complete RPMI was transferred to a 1.5 mL Eppendorf tube, mixed with 75 µL of low melting agarose (0.7% in phosphate buffer, PBS) and distributed onto conventional microscopic slides precoated with normal melting agarose (0.5% in PBS) and dried at 50 °C. After the agarose was solidified (4 °C for 5 min), a second layer of low melting agarose was applied, similar to the previous layer. The slides were then immersed in lysis solution (2.5 M NaCl, 100 mM Na_2_EDTA, 10 mM TrisHCl pH 10.0, containing freshly added 1% Triton ×100 and 10% DMSO) for 1 h at 4 °C and then placed into a horizontal electrophoresis apparatus filled with freshly made buffer (1 mM Na_2_EDTA, 300 mM NaOH, pH 13.0). After 20 min of preincubation (unwinding of DNA), the electrophoresis was run for 20 min at a fixed voltage of 25 V and 300 mA, which was adjusted by raising or lowering the level of the electrophoresis buffer in the tank. At the end of the electrophoresis, the slides were washed three times with neutralization buffer (Tris-HCl 0.4 M, pH 7.5), stained with 50 mL ethidium bromide (20 mg/mL), and kept in a moisture chamber in the dark at 4 °C until analysis. All the above reported steps were carried out under red light to prevent any additional DNA damage.

#### 2.2.7. Comet Detection

The cells were analyzed 24 h after staining at 400× magnification using a fluorescence microscope (Zeiss, Oberkochen, Germany) equipped with a 50 W mercury lamp. The microscopic images revealed circular shapes (undamaged DNA) and “comet-like” shapes in which the DNA had migrated from the head to form a tail (damaged DNA). The extension of each comet was analyzed by a computerized image analysis system (Comet assay II, Perceptive Instruments, Suffolk, UK) that, among several other parameters, gave the “tail moment”, which is considered to be the parameter most directly related to DNA damage. In fact, the tail moment is defined as the product of DNA in the tail and the mean distance of its migration in the tail. Calculation of the extent of DNA damage, which was not homogeneous, was based on the analysis of 100 randomly selected comets from each slide, divided into five classes according to the tail moment (t.m.) values as follows: class 0 (t.m. < 1; no damage), class 1 (t.m. 1–5; slightly damaged), class 2 (t.m. 5–10; medium damage), class 3 (t.m. 10–20; highly damaged), class 4 (t.m. > 20; completely damaged). The overall score expressed in arbitrary units (A.U.) for each slide ranged from 0 (100% of the comets in class 0) to 400 (100% of the comets in class 4) [39]. As internal positive control we generally used hydrogen peroxide, which induces, in human peripheral blood cells after 30 minutes of treatment, a DNA damage of about 150 arbitrary units.

### 2.3. Statistical Analysis

Statistical analysis was carried out using ANOVA based on a linear model, with diet considered the fixed effect [40]. Multiple comparisons were performed using Bonferroni’s range test for all treatments evaluated, except for DNA damage for which the Tukey post-hoc test was used. Significance levels were set at *p* < 0.05 and *p* < 0.01.

## 3. Results

No differences in the feed intake of the three experimental diets were recorded (110 vs. 112 vs. 111 g/d in C, OL, and SeOL rabbits, respectively). Based on these data, the estimated intake of the main bioactive compounds (minerals and antioxidants) was calculated for each dietary group (Table 1).

**Figure 1:**
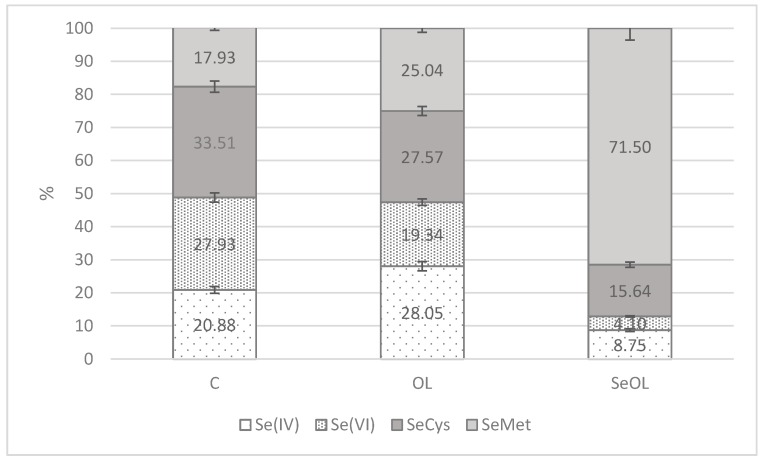
Estimated Se intake (% of different forms) in growing rabbits (n = 10/group) fed the experimental diets. C: control diet, OL: olive leaves supplemented diet, SeOL: Se-enriched olive leaves supplemented diet. SeMet is presented as a light grey bar, SeCys is presented as a dark grey bar, Se (VI) is presented as a bar with squares, Se (IV) is presented as a bar with lines.

The SeOL group had higher intake of Fe and Se, in particular Fe was 1.63- and 1.72-fold higher compared to C and OL, respectively, whereas Se intake was significantly different in all groups (24.03 vs. 1.76 vs. 0.04 μg/d in SeOL, OL, and C, respectively). The inorganic form of Se was more abundant in the OL diet compared to the SeOL diet (Figure 1): 28.05% was constituted of Se (IV) and 19.34% by Se (VI) forms; these values were 8.75% and 4.10%, respectively, in SeOL. The SeMet was the most abundant Se organic form in the SeOL group (71.50% vs. 25.04% and 17.93% in OL and C, respectively), followed by SeCys, which was lower than C and OL (15.64% vs. 33.51% and 27.57% in the SeOL, C, and OL groups).

Regarding the antioxidant intake, the highest quantity of carotenes and vitamin E was found in the SeOL group (mainly the α-tocopherol isoform: 2064.60 vs. 1658.72 and 1446.50 μg/d in SeOL, OL, and C group, respectively), followed by OL and C.

Table 2 reports the mineral profile in plasma. The plasma concentrations of Ca, Mg, and Fe were not significantly different in the experimental groups. On the contrary, Se was 1.6-fold higher in SeOL plasma with respect to the other groups.

The selenium speciation of plasma is reported in Table 3. In agreement with what was found for selenium intake, Se (VI) was higher in the OL group with respect to SeOL and C. However, Se (IV) was not different in the Se-supplemented group (*p* > 0.05). The inorganic form of Se did not show significantly different values in supplemented olive leaves, whereas organic Se had a significantly higher value in the SeOL group. SeMet was the most abundant form in plasma, about 4-fold higher with respect to both OL and C. Conversely, C showed a higher value of MeSeCys with respect to both groups supplemented with olive leaves.

In Table 4, the plasma oxidative status and antioxidant amount are reported. Protein oxidation showed significantly higher values in both groups supplemented with leaves with respect to the control. A slight increase in TBARs, retinol, and α-tocopherol were observed in the OL and SeOL groups, although their values were not significantly different. Other antioxidant compounds (α-tocotrienol, δ-tocopherol, and γ-tocopherol) and the GPx enzyme activities in rabbit plasma were unaffected by the treatments. The dietary intervention resulted in a significant improvement in the reducing power of plasma in the SeOL experimental group, with an increase of about 40%; no differences were recorded between the C and OL groups (Table 4). 

**Figure 2:**
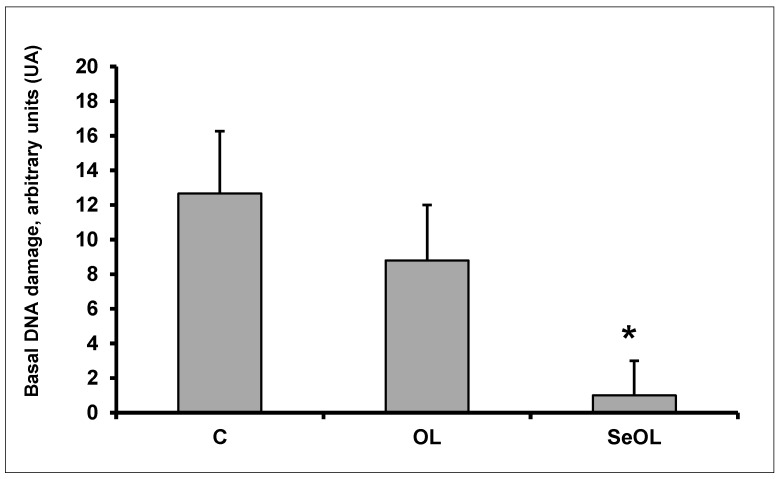
Basal DNA damage in leukocytes isolated from whole blood on rabbits fed diets enriched or not with added olive leaves. C: control diet, OL: olive leaves supplemented diet, SeOL: Se-enriched olive leaves supplemented diet. Values are means ± SD. Means without * differ, *p* < 0.05 (Tukey post-hoc test); (n = 4 rabbits/group).

Figure 2 shows DNA damage in PBMCs isolated from whole rabbit blood. A marked reduction in DNA damage was observed in the SeOL group, where the value was about 9-fold lower compared to the C group (*p* < 0.05). No differences were reported between the C and OL groups.

## 4. Discussion

Selenium consumed in foods exists in a number of organic and inorganic forms, including SeMet (plant and animal sources and supplements), SeCys (mainly animal sources), Se (IV), and Se (VI) (mainly supplements). A body of literature reports that there is no relationship between Se intake and its content in the plasma because this largely depends on the form of Se [41,42] and on the possible antagonistic effects of other mineral compounds [43].

In general, organic forms are absorbed more efficiently than inorganic ones (particularly selenite), with uptake from the gastrointestinal tract of more than 90% for SeMet compared to about 60% for Se (VI) [44]. Furthermore, the chemical form also affects the retention levels in the body over time, as it has been shown that SeMet has a higher storage capacity than the others [45], since it is non-specifically incorporated into proteins; however, the most efficient form seems to be the SeCys because it constitutes the active site of Se-proteins (i.e., GPx, selenophosphate synthetase, thioredoxin reductases, etc. [46]). In addition, organic forms of Se are less toxic and more environmentally friendly than inorganic forms [47].

Furthermore, no antagonistic effect operated by others minerals could be supposed, considered that their concentrations did not change between experimental groups (Ca and Mg) except for the Fe; however, the levels of the latter, although higher in the SeOL diet, maintain values less than the range of competition (20–500 mg/kg of diet; [43]). In addition, no differences in the plasma profile were found for this mineral.

In the present research, sodium selenate nebulized on olive trees (SeOL group) and successively supplemented to rabbits was mainly metabolized by the plant as SeMet. In the OL group, the most representative Se forms were SeCys and Se (IV) at a similar concentration, followed by SeMet and Se (VI). Such a profile was also confirmed in rabbit plasma, where SeMet was the most abundant form in the SeOL group. This is not surprising because SeMet, being non-specifically incorporated into proteins (e.g., hemoglobin, albumin) in place of methionine, is more effective at increasing Se concentrations [46]. The similarity found in the SeCys plasma concentrations was probably due to the incorporation limit and then to the achievement of a metabolic plateau. In agreement with this, it was reported that the central organ of Se metabolism, the liver, first converts inorganic Se to forms incorporated only into selenoproteins (like SeCys), i.e., glutathione peroxidase (GPx) and the Se-transporter selenoprotein P (SEPP1), until an adequate intake range and then incorporates Se in a non-specific manner, i.e., as SeMet, into albumin and other proteins over an unlimited range of intakes [8,48]. Our concurring results demonstrated that no difference was found between groups in terms of plasma Se-GPx activity, according to SeCys values. It is likely that the main form of incorporation in plasma was as SeMet-constituted proteins.

From the metabolic point of view, SeMet is considered to be a less-available metabolic form of selenium than selenite or selenate, since these need only be reduced to selenide to provide selenophosphate, the precursor of selenocysteine [3]. Furthermore, SeMet has no catalytic activity and must be catabolized to an inorganic precursor before entering the available selenium pool. In this regard, Combs et al. [8] compared different studies and reported that inorganic Se (Se (IV) and Se (VI)) induces minimal increases (<20%) in human plasma, with Se concentrations greater than 70 ng/mL [49], while SeMet-containing foods (organic Se) produce considerable plasma Se increases. Similarly, Wastney et al. [48] found that the enteric absorption of Se (200 μg) by healthy humans is 98% for SeMet and 84% for Se (IV). Accordingly, in the present study, the SeMet concentration in rabbit plasma was higher than the others; however, this trend only partially affected the Se metabolic activity because no differences in lipid (TBARs) and enzyme (GPx) oxidative status or the antioxidant profile were found in rabbit plasma.

Along with lipid oxidation, proteins are major targets of reactive oxygen species (ROS) [50], which are considered to be biomarkers of oxidation, often related to different antioxidants. In agreement with this, Delles et al. [51] found lower serum carbonyls associated with a higher antioxidant level. With respect to carbonyls, there were significant differences in plasma, with higher values in both the OL and SeOL groups with respect to the C group. 

Indeed, although olive leaf supplementation ensured high antioxidant intake, no differences were found in the plasma antioxidant content (vitamin E and retinol) or enzyme activity (GPx). Conversely, Ebeid et al. [52] obtained a significant increase in blood serum antioxidative properties when vitamin E or Se were supplemented in a rabbit diet. Casamassima et al. [53] observed, in rabbits on a fat-enriched diet supplemented with dried bay (*Laurus nobilis*) leaves, increased blood antioxidants (retinol and α-tocopherol) and a decrease of TBARs, but in this case, rabbits received the diet for 56 days. The shorter duration of treatment in our study could explain the different results.

Although in our study the single antioxidant compounds in rabbit plasma were unaffected by dietary supplementation, a significant increase in FRAP in the SeOL group suggests that other antioxidant systems could be influenced by Se. For example, selenoprotein P is the main selenoprotein in plasma and contains at least 40% of the whole amount of Se in plasma; it is widely involved in the cellular antioxidant defense [5,6]. Moreover, selenoprotein O may be a redox-active mitochondrial selenoprotein, which interacts with the redox target protein selenoprotein W; this not only seems to interfere with the destruction of H_2_O_2_ pathways but also has a role in immune system function as it affects cytokine production [54]. In this study, we have clearly observed a reduction of basal DNA damage in leuocytes of rabbits fed diets enriched with selenium-treated olive leaves. These results are of interest because they suggest that selenium may improve the health of rabbits and prevent diseases related to DNA damage (cancerogenesis). Several mechanisms may be involved in the selenium induced DNA damage reduction. In particular, SeMet seems to be involved in the DNA repair machinery preventing DNA damage. Similar to our results, Waters et al. [55] observed, using the comet assay, decreased DNA damage both in prostate cells and in peripheral blood lymphocytes in a canine model fed an SeMet-enriched diet. This effect on DNA damage was not associated with GPx activity. Moreover, Se can improve DNA repair enzymes’ abilities, as observed in vitro in the LNCap cell line [56]. Further studies are needed to understand the mechanisms by which selenium reduces DNA damage.

It is well-known that exogenous antioxidants like carotenoids, tocopherols, ascorbyl, and phenolics counteract the activity of the endogenous antioxidative defense (SOD, CAT, GPx, and thiols) [57]. Selenium is not a direct reactive oxygen/nitrogen species scavenger in the redox reaction, but it functions as a cofactor for glutathione peroxidase and so contributes to reducing hydrogen peroxide and peroxides [58]. The present results suggest a synergistic relationship between Se and vitamin E (or other antioxidants furnished by olive leaves) in protecting rabbits against cellular damage by ROS.

## 5. Conclusions

The combination of Se and olive leaves in the diet of growing rabbits caused an increase in plasma Se (SeMet) and ferric reducing power as well as a reduction in basal DNA damage in blood mononuclear cells. This evidence suggests that it is possible use both in animal feed to improve the health status of animals and the quality of meat products [18,30]. This may also be used in the human diet, i.e., in the formulation of new functional foods that could help to meet Se recommended dietary (RDA) requirements and prevent several chronic degenerative diseases that have an oxidative and inflammatory etiology.

## Figures and Tables

**Table 1:** Daily feed intake (g/d) and estimated intake of minerals and antioxidants in growing rabbits (n = 10/group) fed the experimental diets.

Diet Parameters	Unit	Experimental Diets ^1^			*p* Value ^2^	SE ^3^
		C	OL	SeOL		
Daily Feed Intake		110	112	111	0.62	0.67
Minerals ^4^						
Ca	mg/d	673.20	713.05	736.61	0.230	28.25
Mg	“	6.67	6.48	6.36	0.586	2.36
Fe	“	4.95 ^a^	4.68 ^a^	8.08 ^b^	0.048	0.32
Se	µg/d	0.04 ^A^	1.76 ^A^	24.03 ^B^	0.002	1.47
Antioxidants						
α-tocotrienol	µg/d	12.10	7.84	13.32	0.039	3.65
δ-tocopherol	“	19.80	32.48	38.85	0.225	8.65
γ-tocopherol	“	25.30 ^a^	67.20 ^b^	57.72 ^b^	0.020	10.47
α-tocopherol	“	1446.50 ^a^	1658.72 ^a^	2064.60 ^b^	0.021	97.11
ΣVitamin E isomers	“	1512.50 ^a^	1840.16 ^a^	2335.44 ^b^	0.047	201.12
ΣCarotenes	“	5322.90 ^a^	7066.08 ^b^	8721.27 ^c^	0.025	663.45

^1^ C: control diet, OL: olive leaves supplemented diet, SeOL: Se-enriched olive leaves supplemented diet. ^2, a-c^ on the same row differ at *p* < 0.05; ^A, B^ on the same row differ at *p* < 0.01. ^3^ SE: standard error. ^4^ Ca: calcium, Mg: magnesium; Fe: iron, Se: selenium.

**Table 2:** Minerals profile of plasma of growing rabbits (n = 10/group) fed the experimental diets.

Minerals ^4^	Unit	Experimental Diets ^1^			*p* Value ^2^	Pooled SE ^3^
		C	OL	SeOL		
Ca	µg/mL	141.57	183.60	140.80	0.05	24.76
Mg	“	11.10	8.77	10.97	0.12	0.88
Fe	“	5.20	5.90	7.07	0.09	1.24
Se	ng/mL	51.60 ^a^	68.25 ^a^	128.10 ^b^	0.03	33.28

^1^ C: control diet, OL: olive leaves supplemented diet, SeOL: Se-enriched olive leaves supplemented diet. ^2, a, b^ on the same row differ at *p* < 0.05. ^3^ SE: standard error. ^4^ Ca: calcium, Mg: magnesium; Fe: iron, Se: selenium.

**Table 3:** Selenium speciation of plasma of growing rabbits fed the experimental diets.

Se Speciation	Unit	Experimental Diets ^1^			*p* Value ^2^	SE ^3^
		C	OL	SeOL		
Se (IV) ^4^	ng/mL	1.13 ^a^	4.19 ^b^	2.60 ^a^	0.02	0.90
Se (VI) ^5^	“	19.71 ^a^	38.36 ^b^	42.73 ^b^	0.03	10.32
Inorganic-Se	“	20.84 ^a^	42.55 ^b^	45.33 ^b^	0.04	12.60
SeCys ^6^	“	23.22	36.50	26.14	0.14	8.45
SeMet ^7^	“	19.85 ^A^	22.45 ^A^	89.61 ^B^	0.01	12.65
MeSeCys ^8^	“	2.71 ^b^	1.13 ^a^	1.37 ^a^	0.04	0.36
Organic-Se		45.78 ^a^	60.08 ^a^	117.12 ^c^	0.03	20.31

^1^ C: control diet, OL: olive leaves supplemented diet, SeOL: Se-enriched olive leaves supplemented diet. ^2, a-c^ on the same row differ at *p* < 0.05; ^A, B^ on the same row differ at *p* < 0.01. ^3^ SE: standard error. ^4^ Se (IV): selenite. ^5^ Se (VI): selenate. ^6^ SeCys2: selenocystine.^7^ SeMet: selenomethionine. ^8^ MeSeCys: selenomethylselenocysteine.

**Table 4:** Plasma antioxidant compounds and oxidative status of growing rabbits fed the experimental diets.

Oxidative Status	Unit	Experimental Diets ^1^			*p* Value ^2^	SE ^3^
		C	OL	SeOL		
TBARS ^4^	nmol MDA/mL	30.03	42.48	37.08	0.24	7.90
Carbonyls	nmol/mg proteins	0.35 ^A^	2.69 ^B^	2.98 ^B^	0.01	0.74
α-tocotrienol	nmol/mL	0.01	0.01	0.01	0.37	0.01
δ-tocopherol	“	0.45	0.36	0.34	0.30	0.01
γ-tocopherol	“	0.01	0.01	0.01	0.34	0.02
α-tocopherol	“	0.81	0.94	1.44	0.24	0.34
Retinol	“	10.50	13.00	13.83	0.06	2.00
GPx	nmol/min/mg protein	19.81	19.18	19.23	0.12	2.75
FRAP ^5^	μmol/L Fe^2+^	317 ^A^	374 ^A^	444 ^B^	0.01	90.37

^1^ C: control diet, OL: olive leaves supplemented diet, SeOL: Se-enriched olive leaves supplemented diet. ^2 A, B^, on the same row differ at *p* < 0.01. ^3^ SE: standard error. ^4^ TBARS: thiobarbituric reactive substances. ^5^ FRAP: ferric reducing ability of plasma.
